# Evaluating the Efficacy of Monoclonal Antibodies Against a Bioactive Peptide Involved in Alzheimer’s Disease: A Methodological Approach

**DOI:** 10.3390/mps9030074

**Published:** 2026-05-09

**Authors:** Georgina Bonny, Kashif Mahfooz, Sara Garcia-Rates, Sibah Hasan, Susan Adele Greenfield

**Affiliations:** Neuro-Bio Ltd., Building F5, Culham Science Centre, Abingdon OX14 3DB, UK; georgina.bonny@neuro-bio.com (G.B.); kashif.mahfooz@neuro-bio.com (K.M.); sibah.hasan@neuro-bio.com (S.H.); susan.greenfield@neuro-bio.com (S.A.G.)

**Keywords:** monoclonal antibody, T14, validation, Alzheimer’s disease, therapeutic

## Abstract

Antibody treatment for Alzheimer’s disease is an evolving therapeutic strategy that ensures high affinity and specificity to the target antigen; however, current approaches have proven only partially successful. A 14-mer peptide, T14, is twice as high in Alzheimer’s brains and has been identified as a primary driver in the neurodegenerative process. Previously, the polyclonal antibody Ab-19 was shown to be as effective as the T14 receptor blocker (NBP-14) in reducing the toxic calcium influx in PC12 cells. The aim of this study was to establish a thorough validation process in order to evaluate the efficacy of respective anti-T14 monoclonal antibodies in T14 detection and rescuing potential from T14-induced toxicity in PC12 cells. Subsequently, we assessed the binding affinity of the most promising antibody, THK-117, via quantitative indirect conjugated T14 ELISA assays. The level of efficacy shown proved to be comparable to the polyclonal antibody, yet with the additional advantage of robust manufacturing reproducibility and high binding specificity toward the T14 epitope. With a notably low EC50, THK-117 can be viewed as a promising candidate for humanization, offering a strong potential as a therapeutic monoclonal antibody for the treatment and prevention of Alzheimer’s disease.

## 1. Introduction

Over the last few decades, multiple pathophysiological mechanisms have been proposed to explain neurodegeneration in Alzheimer’s disease (AD). One hypothesis suggests that dysregulation of the acetylcholinesterase-derived 14-mer peptide T14 may act as an upstream contributor to AD pathology [1]. T14 has been reported at elevated levels (two-fold) in post-mortem AD brain tissue [1] and has been shown to interact with the α7 nicotinic acetylcholine receptor (α7 nAChR), influencing calcium signalling pathways [1]. During early development, this peptide has been associated with trophic signalling processes regulated by mTOR [1]. Importantly, independent evidence has demonstrated that acetylcholinesterase-derived C-terminal peptides, including the longer T30 sequence that contains the T14 region, can activate α7 nAChR signalling and downstream mTOR pathways, supporting the broader functional relevance of this peptide family beyond our original observations [2].

T14 levels decline with age [1]; however, in the adult brain, pathological reactivation—potentially driven by oxidative stress [3]—may contribute to dysregulated calcium signalling. In aged neurons, reduced calcium buffering capacity [1] may further exacerbate this process, leading to cytotoxicity and neuronal cell death [4], believed to drive neurodegeneration. Furthermore, these downstream toxic cascades have been proposed to include associations with β-amyloid accumulation and subsequent tau hyperphosphorylation [1].

Importantly, α7 nAChR signalling has been independently implicated in calcium-dependent neurotoxicity and synaptic dysfunction [5,6], supporting the biological plausibility of receptor-mediated calcium dysregulation pathways in neurodegeneration. More broadly, peptide–receptor signalling systems are increasingly recognized as modulators of neuronal homeostasis and disease-associated cascades across multiple neurodegenerative conditions. In parallel, rigorous antibody validation frameworks have been widely established in biomedical research to ensure specificity, reproducibility, and translational relevance [7,8]. These approaches typically combine biochemical detection of the target, functional testing in relevant cell systems, and quantitative assessment of binding strength.

Previous research has demonstrated that the anti-T14 polyclonal antibody (pAb) Ab-19 exerts comparable rescuing potential to the T14 receptor blocker NBP-14 at reducing calcium influx in PC12 cells [9]. This in vitro attenuation of the T14 toxic effect acts as a clear indicator of the pAb therapeutic potential based on the T14 AD hypothesis. Despite these promising findings, the clinical application of pAbs presents significant challenges. Ab-19 cannot be humanized, limiting its suitability for therapeutic use due to the risk of immunogenicity and anti-drug antibody responses (ADAs) [8]. Furthermore, the inherent variability and lack of consistency in pAb production pose further concerns regarding reproducibility and efficacy [8]. To develop this, monoclonal antibody (mAb) clones named THK-117 and THK-104 were produced for comparison, with the aim of future therapeutic use.

This study establishes antibody target validation methodology by outlining a clear sequence to identify highly specific interactions for human therapeutic use. The antibody validation process integrates both diagnostic and therapeutic criteria, providing a framework for evaluating future antibody candidates, including those developed for therapeutic applications based on the T14 hypothesis. This study initially used Western blot and immunoneutralization assays to assess T14 detection in rat and AD human brain samples using the mAbs. This was followed by cell-based assays, involving calcium fluorometry in PC12 cells, considered to be the most direct measurement of T14 toxicity. Lastly, the antibody-peptide interaction was quantified and evaluated through an indirect conjugated T14 ELISA, establishing the binding profile and suitability for humanization.

## 2. Materials and Methods

### 2.1. Ex Vivo Rat Brain Slices

Brain samples were obtained from Male Wistar rats (Charles River, Harlow, UK) on postnatal day 7 (P7) and used for Western blot protein detection and validation. Procedures were carried out in line with the Home Office UK regulations (Schedule 1) and in compliance with requirements of the UK Animals (Scientific Procedures) Act 1986 and associated guidelines. Slices were taken at 400 µm thick using a Leica VT100S vibratome in oxygenated cutting solution containing 2.4 CaCl_2_, 1.2 KH_2_PO_4_, 20 NaHCO_3_, 5 KCl, 120 NaCl, 2 MgSO_4_, 10 glucose, 6.7 HEPES salt and 3.3 HEPES acid in mmol. Slices were hemisected with the cortex removed and the Hippocampus (HPC) dissected at stereotaxic coordinates: −2.12 to −6.30 mm from Bregma. Slices were then homogenized in lysis buffer and stored at −80 °C.

### 2.2. Human Clinical Samples

Human Hippocampal brain tissue was provided by Oxford Brain Bank at John Radcliffe Hospital with an ethics approval under Oxford Brain Bank’s generic REC approval on behalf of the Chancellor, Masters and Scholars of the University of Oxford. Approval number: 15/SC/0839. Reference number: 07/Q1605/16. Approval date: 31 July 2019. This complied with the Human Tissue Act 2004, Human Tissue Regulations 2007, Health and Safety laws and other applicable laws relevant to post-mortem tissue. The privacy rights of human subjects have been observed, and informed consent was obtained for experimentation with human subjects. The samples selected included Hippocampal brain tissue at Braak II and VI stages of Alzheimer’s disease, from females aged 88 and 92.

### 2.3. Monoclonal Antibody Generation

The monoclonal antibodies (Abcam plc, Cambridge, UK) used in this study were generated in rabbits by immunizing them with the T14 peptide (Genosphere Biotechnologies, Paris, France). Rabbit serum was subsequently screened against the immunogen and full-length acetylcholinesterase. Splenocytes were isolated from the immunized rabbits for further analysis. B-cells were enriched and subjected to repertoire sequencing and bioinformatic analysis to select the recommended clones and generate a lineage map. Up to 50 clones were generated and screened against T14 by synthesizing heavy and light chains, creating mammalian expression vectors and performing binding assays with T14 peptides and monomeric alpha-synuclein. Clone selection was based on binding specificity to the T14 peptide and the absence of cross-reactivity with unrelated peptides. THK antibodies were produced and purified from a single clone after transient transfection, Protein A purification and QC testing.

### 2.4. Western Blot Antibody Detection and Immunoneutralization

In total, 5 µg of protein was combined with 10× Bolt^TM^ Sample Reducing Agent (catalogue number: B0009, Invitrogen, Waltham, MA, USA) and 4× Laemmli Sample buffer catalogue number: 1610747, BioRad, Hercules, CA, USA), heated for 10 min at 60 °C before being loaded into a TGX gel (BioRad 4–20%, catalogue number: 4561096, BioRad, Hercules, CA, USA). Electrophoresis was used to separate the proteins in each sample for 30 min per gel at 35 mA in 10× Trig-Glycine-SDS running buffer (catalogue number: 4561096, Fisher Scientific, Leicestershire, UK) before being transferred at 200 mA for 90 min onto polyvinylidene fluoride (PVDF) membrane (0.2 µM pore size, catalogue number: 160117 BioRad, Hercules, CA, USA) using 10× Tris-Glycine transfer buffer (catalogue number: 1610771, BioRad, Hercules, CA, USA) and methanol. The membrane was blocked for 1 h in 10 mL of a 5% BSA solution dissolved in Tris-buffered saline solution (TBS) and 0.005% Tween-20 (TBS-T) before the monoclonal anti-T14 antibody (THK-104 or THK-117) was added at 2:5000 in blocking solution and left to incubate overnight at 4 °C on a rocker. Anti-T14 monoclonal antibodies were custom-made by Abcam. The membrane was washed 3 times for 5 min each in TBS-T before incubation with a custom-made secondary goat anti-rabbit IgG H+L Horseradish peroxidase (HRP) conjugated antibody (1:10,000, catalogue number: G21234, Thermo Fisher Scientific, Swindon, UK) in blocking solution at room temperature (RT) for 1 h on a rocker. The wash process with TBS-T was repeated 3 more times with an additional wash using TBS for 5 min. The proteins were visualized using a chemiluminescence-based detection kit, ECL, and a CCD camera (GBox, Syngene, Cambridge, UK) gel imaging system. The protein bands were analyzed using ImageJ (version 1.54, National Institutes of Health, Bethesda, MD, USA).

### 2.5. Antibody Detection of T14 Validation

The monoclonal antibodies were pre-incubated with a 15 AA N-cys-T14 peptide (Genosphere Biotechnologies, Paris, France) for 3 h at RT on a roller at 20-times excess for a concentration of 2:5000 diluted in 5% BSA to ‘block’ the antibodies. The membrane was then incubated for 1 h at RT with the blocked antibodies or the unblocked monoclonals as a comparison for validity. The same secondary Goat anti-rabbit IgG antibody was subsequently added (1:10,000) and diluted in 5% BSA for a 1 h incubation at RT on a rocker before being washed and imaged. The data was analyzed using ImageJ for visual detection and confirmation of antibody validation from peptide blocking. Data was quantified and compared as a percentage of the unblocked antibody.

### 2.6. PC12 Cell Culture

PC12 cells are a pheochromocytoma cell line derived from the rat adrenal medulla, resulting in a diverse, easily cultured cell line model with a suitable application to study neuronal functions [10], purchased from Sigma (Merck kGaA, Darmstadt, Germany, 88022401). The cells were maintained in type IV collagen coated (2 mg/cm^2^, Sigma, Merck kGaA, Darmstadt, Germany C5533) 100 mm dishes (Corning, Somerville, MA, USA, StarLab CC7682-3394) using complete Dulbecco’s Modified Eagle Medium (DMEM) with high glucose, 10% heat-inactivated horse serum (HS) and 5% Fetal Bovine serum (FBS), 0.1 mg/mL Penicillin/Streptomycin and 2.5 µg/mL Amphotericin B. Amphotericin B was included at low concentration as part of standard antifungal supplementation during extended PC12 culture. We acknowledge its potential effects on membrane permeability; however, it was present in all experimental conditions, including controls, and therefore is unlikely to account for the observed differences between groups. The medium was changed every 2–3 days and kept at 37 °C. Cells were mechanically detached using a cell scraper and split through a syringe using passages 14 through 28.

### 2.7. Calcium Fluorometry

In total, 100 µL of undifferentiated PC12 cells was plated at a 15% confluency on a collagenated black clear-bottom 96-well plate and left for 48 h at 37 °C in complete DMEM. Sterile water was added to the outermost wells to retain humidity and minimize the plate effect. On the day of the experiment, in low light conditions, Fluo-8 assay buffer was prepared according to the manufacturer’s protocol (Abcam, Cambridge, UK, 112, 128), adding 7.5 µL of Fluo-8 dye to 7.5 mL of Hanks Balanced Salt Solution (HHBS) and 0.1% Pluronic F127 Plus assay buffer. In total, 100 µL of growth medium was removed from the plate and replaced with 70 µL of the treatments and dye. These included a Control, T14 dissolved in 5% Acetonitrile, the custom-made monoclonal antibodies: THK-117, THK-104, the pAb A6, GAPDH and PNU120596. The polyclonal antibody (pAb) serves as a validated biological positive comparator for T14 neutralization (in cells), previously shown to rescue calcium influx in the same system [9]. A commercial monoclonal anti-T14 antibody would be valuable for future benchmarking but is not currently available with validated specificity for this peptide. After treatment, the plate was then incubated at 37 °C for 30 min, followed by 80 min in the dark at RT. The treatments added were dependent on the passage number of the cells, such as 250 nM T14 for passages 18–19 or 50 nM T14 for passages 25–27. The fluorescence was read at 490/525 nm using a fluorescence plate reader (Fluostar, Optima, BMG Labtech, Ortenberg, Germany), where 50 µL Acetylcholine (128 µM) was injected into each well, acting as an agonist of the α7 nAChRs and the fluorescence was recorded. For data analysis, the average baseline reading was subtracted from the average maximum reading following the Acetylcholine injection. The data was pooled from multiple experiments and expressed as a percentage of the untreated control average, with statistical analysis of the log-transformed data performed using GraphPad Prism version 10.2.0 software.

### 2.8. T14 Indirect ELISA

The binding profile of free T14 was depicted using a free T14 indirect Enzyme-linked immunosorbent assay (ELISA). Fresh T14 was reconstituted in 5% Acetonitrile and diluted to 1 µg/mL before being loaded onto a 96-well NUNC^TM^ Micro-Well^TM^ plate (Thermo Fisher Scientific) and maintained at 4 °C on a shaker overnight. The wells were then washed with 200 µL PBS-T (Phosphate-Buffered Saline + 0.1% Tween-20) three times and then blocked with 5% BSA for 120 min. The wells were washed, and the custom-made rabbit anti-T14 monoclonal antibody THK-117 was added at 12-point, 2-fold serial dilutions in 1% BSA dissolved in PBS, starting at 240 µg/mL. This was incubated in the 96-well plate for 90 min at RT. Following this, the plate was washed three more times, and the HRP-conjugated goat anti-rabbit IgG (1:10,000, Thermo Fisher Scientific) was added and left on the shaker at RT in the dark for a further 60 min. The wells were washed for a final time before adding the 1-Step^TM^ TMB substrate (Thermo Fisher Scientific) for 25 min in the dark before the reaction was stopped using 2M H_2_SO_4._ The absorbance was then measured at 450 and 540 nm using a ClarioSTAR (BMG Labtech).

### 2.9. BSA-T14 Indirect ELISA

Monoclonal antibody dose response EC_50_ values were calculated using T14 and T14 conjugate indirect ELISAs, then compared. In total, 100 µL of 1 µg/mL BSA-conjugated T14 (Abcam) was plated in a 96-well NUNC^TM^ Maxisob Micro-Well^TM^ (Thermo Fisher Scientific) ELISA plate and maintained on a plate shaker overnight at 4 °C. Wells were washed with 200 µL PBS-T (0.1% Tween-20) three times, then blocked with 3% BSA for 120 min at RT on a plate shaker. The washing process was repeated, and 100 µL of the custom-made primary monoclonal antibody THK-117 was added in 15-point, 3-fold serial dilutions starting at 10 µg/mL and left for 90 min at RT on the plate shaker. After washing, 100 µL of HRP-conjugated goat anti-rabbit IgG (1:10,000, Thermo Fisher Scientific) was added and left for 60 min at RT in the dark on a plate shaker. Wells were washed, and 100 µL 1-step^TM^ TMB substrate (Thermo Fisher Scientific) was added to each well, then left in the dark for 10 min, followed by the addition of 100 µL 2M H_2_SO_4_ stop solution. The absorbance was measured at 450 and 540 nm using a ClarioSTAR (BMG Labtech) plate reader.

### 2.10. Biotinylated-T14 Indirect ELISA

The Indirect ELISA process was repeated using a precoated 96-well Streptavidin Pierce^TM^ with SuperBlock^TM^ blocking buffer plate (Thermo Fisher Scientific) for Biotinylated-T14. The plate was washed 3 times with 150 µL PBS + 0.1% Tween-20. Subsequently, 100 µL of 1 µg/µL Biotinylated-T14 (Abcam) was plated per well and incubated for 1 h at 37 °C. Wells were washed and then blocked with 150 µL PBS + 1% BSA + 0.05% Tween-20 for 2 h at RT on a plate shaker. The washing process was repeated, and 100 µL 15-point, 3-fold serial dilutions of THK-117 were added to the plate and left for 2 h on a plate shaker at RT. The plate was washed as above, and 100 µL of secondary antibody HRP-conjugated goat anti-rabbit IgG (1:10,000, Thermo Fisher Scientific) was added, diluted in PBS + 0.1% BSA + 0.05% Tween-20 for 1 h at RT on the plate shaker in the dark. After repeated washes, 70 µL 1-step^TM^ TMB substrate (Thermo Fish Scientific) was added and left for 10 min in the dark on the shaker, and 35 µL 2M H_2_SO_4_ stop solution was then added. The absorbance was read at 450 and 540 nm using a ClarioSTAR (BMG Labtech) plate reader.

### 2.11. Statistical Analysis

For Calcium fluorometry data, a one-way ANOVA followed by Dunnett’s post hoc test was used for multiple comparisons against the control group. Where appropriate, comparisons against the T14 condition were also included. Data from the indirect T14 ELISAs was also analyzed using GraphPad Prism (version 10.6.1, GraphPad Software, Boston, MA, USA) with statistical analysis of EC_50_ values calculated by normalizing the log-transformed data, with a nonfit linear regression comparing Biotinylated-T14 and BSA-T14. Known concentrations of the monoclonal antibodies were plotted against the respective optical density. Statistical significance was taken at * *p* value < 0.05 for all tests, with graphs presented as Mean ± Standard Error of Mean (SEM).

## 3. Results

### 3.1. Western Blot Detection and Immunoneutralization for Peptide Validation

**Figure 1:**
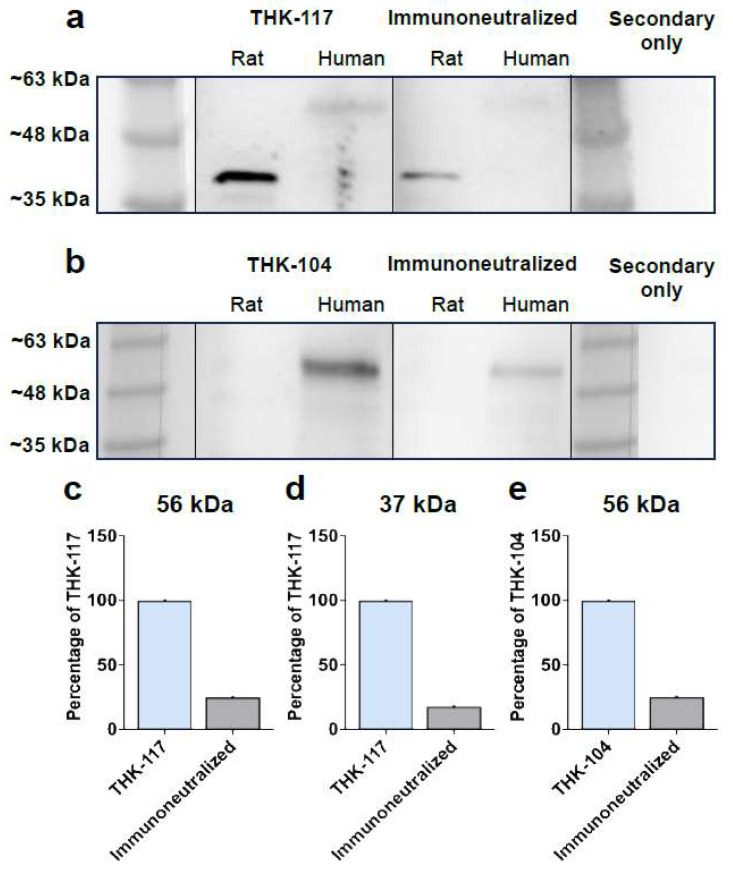
Western Blot Detection and Immunoneutralization for peptide validation. Chemiluminescent T14 signal detection using (**a**) THK-117 and (**b**) THK-104 monoclonal antibodies in P7 Rat and Human Hippocampal Braak stage II brains. (**a**,**b**) The immunoneutralized THK-117 and THK-104 are shown in the middle panel, followed by a secondary-only control on the right panel. Bands quantified as a percentage of the unblocked monoclonal antibody at (**c**) ~56 kDa for THK-117 in human Braak stage II brain, (**d**) ~37 kDa for THK-117 in P7 rat brain and (**e**) ~56 kDa for THK-104 in human Braak stage II brain.

The two anti-T14 monoclonal antibodies THK-117 and THK-104 were selected for T14 peptide detection using a Western blot as the initial validation step. THK-117 was shown to detect T14 at 56 kDa in human HPC Braak stage II samples and at 37 kDa for P7 rat brain samples (Figure 1a left panel). Previous studies have reported that antibodies targeting the α7 nicotinic acetylcholine receptor can detect multiple immunoreactive bands on Western blots, including the canonical ~56 kDa band as well as lower molecular weight bands, depending on species, tissue preparation and antibody clone [11]. Monoclonal and polyclonal α7 nAChR antibodies have been shown to label bands between ~40 and 60 kDa [11], with additional lower bands reported under certain experimental conditions. Therefore, the detection of a ~37 kDa band in rat tissue was further assessed using immunoneutralization to confirm specificity. T14 levels were reduced when immunoneutralized (Figure 1b middle panel), with the ‘blocked’ antibody signal reducing from 100% to 24.9% at 56 kDa (Figure 1c) and from 100% to 17.8% at 37 kDa (Figure 1d). Similarly, the THK-104 antibody detected T14 at 56 kDa in human HPC Braak stage II, although in P7 samples, the bands were not prominent enough to be quantified (Figure 1b, left panel). The ‘blocked’ THK-104 resulted in a reduced T14 detection (Figure 1b, right panel) with a reduction to 25.3% (Figure 1e). There were no bands produced in the secondary only control (Figure 1a,b, right panel).

### 3.2. Reversal of T14 Toxicity with THK-117 and THK-104 In Vitro

**Figure 2:**
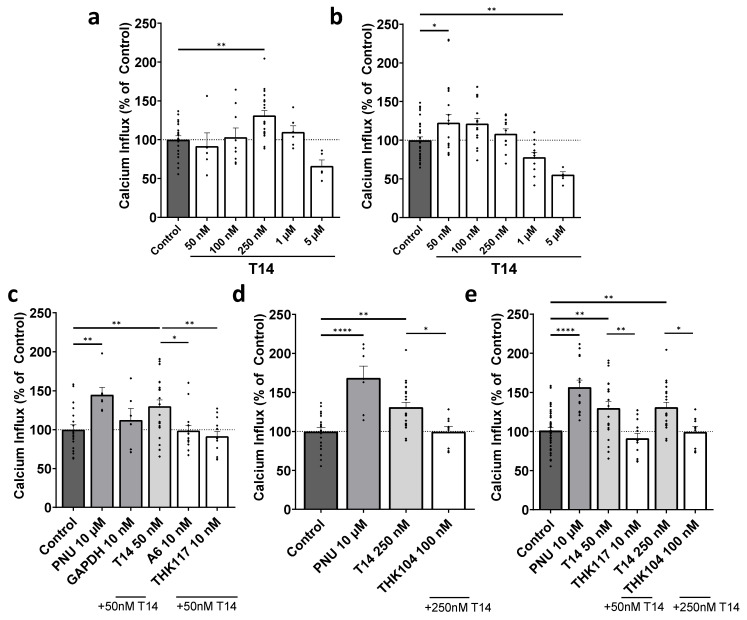
Reversal of T14 toxicity by THK-117 and THK-104 in vitro. Calcium influx in PC12 cells at passages (**a**) 18–19 and (**b**) 25–27 in response to increasing T14 concentrations. Each dose was tested in 6–20 replicates to assess the response. (**c**) Inhibition of calcium influx induced by 50 nM T14 (passages 18–19) with polyclonal A6 and monoclonal THK-117 antibodies. (**d**) Inhibition of calcium influx induced by 250 nM T14 (passages 25–27) with monoclonal THK-104. (**e**) Comparison of both monoclonal antibodies on T14-induced calcium influx (both 50 nM and 250 nM) (mean ± SEM). PNU-120596 (PNU) serves as a positive allosteric modulator control, and GAPDH as a negative control producing no rescuing effect. Calcium influx is represented as a percentage of untreated control cells. One-way ANOVA (“factor” dose) with Dunnett’s post hoc tests were used to determine significance relative to control (100%) or relative to T14 (at 50 nm for panels (**c**,**e**), and 250 nm for panels (**d**,**e**)), where appropriate. *: *p* < 0.05, **: *p* < 0.01, ****: *p* < 0.0001.

THK-117 and THK-104 antibody validation was continued by comparing their efficacy in PC12 cells by reducing the toxic effects caused by T14 administration. T14 dose–response was measured by calcium fluorometry, demonstrating T14 exerted the greatest toxicity when applied at 250 nM at passages 18–19 with an average calcium influx of 131.2% (Figure 2a, one-way ANOVA, F = 6.089, *p* = 0.0001, R^2^ = 0.3329, Dunnett’s post hoc test, *p* = 0.0024). However, when repeated in passage 25–27 cells, 50 nM resulted in the greatest calcium influx, increasing to 122.5% on average (Figure 2b, one-way ANOVA, F = 7.654, *p* < 0.0001, R^2^ = 0.2983, Dunnett’s post hoc test, *p* = 0.0469). This dictated the T14 concentration applied to the cells in each assay (Figure 2c–e). The dose-dependent experiments (Figure 2a,b) consistently showed that higher concentrations of T14 resulted in a decrease in calcium influx, suggesting a desensitization of receptor activity.

The polyclonal antibody, previously validated for T14 neutralization in PC12 systems, was used as a functional positive comparator. The rescuing potential of the A6 polyclonal and THK-117 monoclonal antibodies was compared by their ability to reduce T14-induced calcium toxicity. THK-117 was shown to significantly reduce the calcium influx caused by 50 nM T14 by 38.5% (Figure 2c, 91.5 ± 18.4%, one-way ANOVA, F = 5.652, *p* = 0.0002, R^2^ = 0.2711; Dunnett’s post hoc test, *p* = 0.0023) at a comparable rate to A6 which decreased the influx by 31.1% (98.9 ± 21.2%; Dunnett’s post hoc test, *p* = 0.0134). When the negative control GAPDH was applied with 50 nM T14, no significant rescuing effect occurred compared to T14 (Dunnett’s post hoc test; *p* = 0.6234). The inhibition of THK-104 monoclonal antibody was investigated and shown to significantly reduce the 250 nM T14-induced calcium influx by 31.5% (Figure 2d, 99.65 ± 16.75%, one-way ANOVA, F = 5.652, *p* = 0.0002, R^2^ = 0.2711, Dunnett’s post hoc test *p* = 0.0143). Furthermore, when both of the mAbs were compared, THK-117 at 10 nM showed a more significant calcium influx reduction compared to THK-104 at 100 nM (Figure 2e, one-way ANOVA, F = 12.01, *p* < 0.0001, R^2^ = 0.3532, Dunnett’s post hoc tests *p* = 0.0013, *p* = 0.0323, respectively).

### 3.3. Binding Profile of THK-117 Using Indirect Free T14, BSA-T14 and Biotin-T14 ELISA

**Figure 3:**
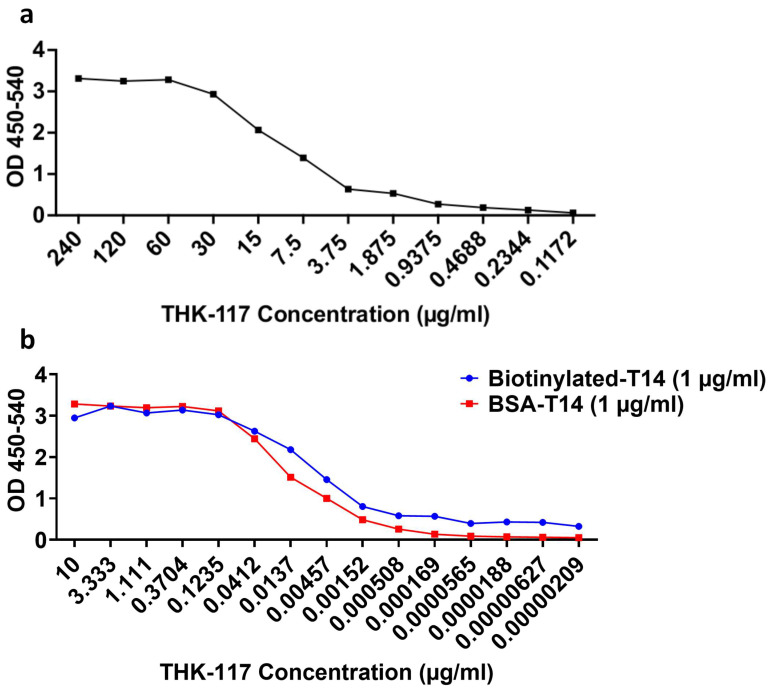
The binding profile of THK-117 using Indirect Free T14, BSA-T14 and Biotin-T14 ELISA. The binding profile of THK-117 monoclonal antibody when bound to (**a**) free T14 or (**b**) BSA-T14 and Biotin-T14. T14 variants were added at 1 µg/mL diluted in 5% Acetonitrile. THK-117 was serially diluted (**a**) 2-fold and (**b**) 3-fold. Binding affinity presented as Optical density 450–540 against known mAb concentrations. Data was transformed and normalized before being analyzed using a nonfit linear regression curve comparing the inhibitor and the normalized response on a variable slope.

Following the treatment data, THK-117 was shown to have superior therapeutic application and was characterized by producing a binding profile in order to calculate the EC_50_ value. As the THK-117 concentration was reduced from 240 µg/mL for a 12-point, 2-fold serial dilution, the binding rate simultaneously decreased and plateaued (Figure 3a), producing a binding profile EC_50_ of 9.535 µg/mL. This was repeated using BSA and Biotin-conjugated T14. A similar binding profile was observed (Figure 3b) with 15-point, 3-fold serial dilutions resulting in a plateau at 0.00051 µg/mL compared with unconjugated T14 gently plateauing at 1.88 µg/mL. The conjugated T14 peptide ELISAs resulted in significantly lower EC_50_ values. Biotinylated-T14 produced an EC_50_ value of 0.007811 µg/mL, and BSA-T14 produced an EC_50_ value of 0.01328 µg/mL.

## 4. Discussion

### 4.1. Developing Therapeutics

Alzheimer’s disease therapeutics are a leading priority for society, highlighted by a recent £160 million pledge to support dementia research in the UK alone [12]. Antibodies are a developing therapeutic strategy commonly used for cancer, autoimmune diseases and chronic inflammatory diseases, with more recent application in neurodegenerative diseases, including AD. These disease-modifying therapies [13] are engineered to ensure a high specificity and high binding affinity, minimizing off-target adverse effects from skin rashes to more serious systemic responses [8], which would compromise clinical trial outcomes, affecting the overall efficacy. Furthermore, high affinity is imperative to ensure adequate dosage through the Blood–Brain Barrier, which is a substantial challenge in neurodegenerative antibody administration. Anti-amyloid antibodies such as Lecanemab have been designed to overcome this boundary using transcytosis mechanisms [14]. Despite this, current anti-amyloid antibodies show limited clinical benefit because amyloid accumulation, although detectable in early stages of AD [15], develops after pathogenic drivers such as T14 [1].

### 4.2. Rationale of Methodology

A standardized methodology for antibody validation is an essential scientific tool for furthering therapeutic development, which currently has insufficient guidelines and subjective criteria [7]. A rigorous validation process is necessary to ensure antibodies are specific, selective and reproducible in order to maximize therapeutic output whilst maintaining high standards in health care. Without a thorough validation process, antibody production can result in inconsistent findings, leading to inaccurate characterization of antibodies, wasting money and resources. The methodology needs to be thorough whilst achievable to be universally accepted and attainable. This should include a Western blot for peptide detection and application suitability [16] with a clear band at the predicted molecular weight, followed by immunoneutralization to demonstrate the immunogen specificity. Functional relevance should be demonstrated using in vitro cell-based assays that confirm a biologically meaningful effect, supporting progression through the validation pipeline [7,17]. Reproducibility should also be assessed under varying experimental conditions. Further data should be provided using conjugated peptide ELISA assays. This is a crucial step in therapeutic antibody development, particularly for antibodies intended for humanization, as it enables assessment of low EC_50_ values relevant to future clinical application [18].

### 4.3. T14 Peptide Detection and Immunoneutralization Using THK-104 and THK-117

The antibody validation process typically begins with Western blot detection to assess specificity and selectivity. Experimental conditions can influence reproducibility and therefore require careful optimization. In this study, a 5% BSA blocking solution was used for T14 detection, as it provided reliable signal sensitivity [19]. The same blocking condition was maintained for ELISA experiments, including the biotinylated T14 assay, to ensure consistency across methods.

THK-117 detected a clear band in the P7 rat brain at ~37 kDa and at ~56 kDa in the human brain, consistent with previous findings indicating that T14 can be detected at distinct molecular weights, potentially reflecting different isoforms. Commercial α7 nAChR polyclonal antibody (ab23832) detects the canonical ~56 kDa band as well as additional lower molecular weight bands at 37 kDa, which can disappear with stringent denaturation as noted in the datasheet 23832 [20]. Additionally, the monoclonal α7 nAChR antibody M220 has been reported to recognize two bands at ~56 kDa and ~44–46 kDa in rat hippocampal homogenates, with the lower band sensitive to changes in blocking conditions [21]. The observation that THK-117 detects a ~37 kDa band in rat brain and a ~56 kDa band in human hippocampal tissue is therefore consistent with previous reports indicating that multiple immunoreactive bands can appear when probing α7 receptors by Western blot. This may reflect antibody binding to the full-length receptor, alternatively processed forms (including splicing variants, post-translational modifications or degradation products), or epitope-specific recognition by individual antibody clones. In another study, different α7 antibodies (targeting different α7 amino acids) showed variable banding patterns in the human thalamus membranes and in rat brain membranes, including a single band of 62 kDa (which was altered by blocking conditions) and a doublet of 53–54 kDa [22]. Altogether, these studies demonstrate that not all α7-immunoreactive bands correspond to the canonical receptor size; additional bands have been shown in some studies to be condition-dependent and reduced or eliminated by denaturing/blocking, supporting their relationship to the receptor rather than nonspecific background. In the present study, the marked reduction in the ~37 kDa signal following immunoneutralization provides independent evidence of specificity, supporting the interpretation that this band represents a biologically relevant T14-associated signal in rat tissue. Experiments were performed using both rat and human brain samples to compare antibody performance, alongside secondary-only controls and a previously characterized polyclonal antibody [9] shown to detect T14 in both species. THK-117 successfully detected T14 in both mammalian samples; however, detection using the THK-104 monoclonal antibody was limited to the human brain sample, indicating reduced reliability and a more restricted application for T14 detection. An independent validation method was implemented using immunoneutralization to confirm specificity and appropriate T14 band assignment. Immunohistochemistry has demonstrated the effectiveness of immunoneutralization [23], supporting its use for T14 band confirmation.

To avoid misinterpretation, we note that the T14 signalling pathway and its antibody-based modulation remain an emerging area of investigation. While previous work from our group has demonstrated antibody-mediated antagonism of T14 in vitro [9], the present study provides a more systematic validation framework, incorporating monoclonal antibodies and multiple complementary validation approaches. Independent replication in additional laboratories will be essential to confirm and extend these findings.

Following immunoneutralization with a 20-fold excess of T14 peptide, signal detection was markedly reduced, confirming antibody specificity. This represents a key step in antibody characterization and supports its use in future studies.

### 4.4. PC12 Cells as Alzheimer’s Models

The PC12 cell line, derived from rat adrenal pheochromocytoma [1], is a well-established cell line used for AD research, including therapeutic design, due to its ability to synthesize, store and release catecholamine neurotransmitters [24] including dopamine and Acetylcholine, known to be key in AD development [1]. Owing to this, they are described as providing a ‘window’ into the brain [25] perfect for conducting studies on neuroprotection, neurosecretion, neurotoxicity, neuroinflammation and synaptogenesis [26], explaining why they are frequently used for in vitro drug screening [1]. Furthermore, there are high amounts of the α7-nACh receptors to which T14 selectively binds [27] within the PC12 cells, believed to influence calcium-dependent signalling, the cholinergic anti-inflammatory axis, neurotransmitter release and dendritic plasticity [10] highlighting the relevance of using PC12 cells for AD monoclonal treatment application.

### 4.5. In Vitro Validation Using Calcium Fluorometry

Although Ab-19 demonstrated promising in vitro efficacy, its therapeutic potential is limited by the inherent variability of polyclonal antibody production and the associated risk of anti-drug antibody responses (ADAs) [8]. To address the limitations, anti-T14 mAb clones were developed and rigorously validated to assess their specificity, consistency, and suitability for therapeutic application. Following this validation process, selected mAbs may be suitable for humanization via complementary-determining region (CDR) grafting [8], thereby reducing immunogenicity while maintaining stability and target specificity.

Here, we investigated the rescuing potential of THK-117 and THK-104 in PC12 cells to further support antibody validation whilst confirming a meaningful biological effect based on the T14 hypothesis [28]. Calcium influx was used as the primary cell-based parameter, as previous research has shown it to be a direct measure of T14 toxicity [29]. Given that calcium cytotoxicity has previously induced cell death by up to 70% [1,9], this represents a key validation step.

This cell death occurs as a consequence of T14 selectively binding to the α-7 nAChR [1] in PC12 cells. This receptor-specific mechanism is further supported by reduced bioactivity observed in the striatum compared to the substantia nigra, a region rich in nicotinic receptors but lacking α7-nAchRs, unlike the substantia nigra [30].

T14 binds to an allosteric site on the α7-nAchR, mediating the influx of intracellular calcium levels [1], which results in oxidative stress and cytotoxicity [29]. This is further supported by studies showing that overexpression of this receptor in PC12 cells leads to increased or prolonged calcium influx [5].

However, it was important to assess the impact of passage number on calcium-induced cytotoxicity to ensure reproducibility when comparing both mAbs. Lower passage numbers showed maximal calcium influx at 250 nM T14, whereas at higher passages (~25), 50 nM produced the most consistent and robust response. These observations informed the selection of T14 concentrations used in each antibody assay.

Both mAbs significantly reduced calcium influx, indicating effective binding to T14 and inhibition of receptor-mediated signalling. Their effects were comparable to those observed with the previously characterized polyclonal antibody A6, supporting their functional relevance. However, THK-117 demonstrated a more pronounced rescuing effect, further supporting its biological relevance and justifying its selection for subsequent characterization.

### 4.6. Binding Profile of THK-117 Using T14 Indirect ELISA

The next substantiation stage was an indirect T14 ELISA, chosen for its acute sensitivity and accessibility [31] in high-throughput screening. This quantitative assay is able to measure binding affinity [32] and help determine antibody humanization suitability using EC_50_ values [33] to provide reproducible numerical data for antibody comparison. This assay determines the detection limits of the antibody [34,35] which is crucial data for any antibody with a therapeutic humanization aim, whilst demonstrating the causal relationship present. Additionally, the binding profile indicates specificity in the antibody–antigen binding complex [35], critical for minimizing off-target adverse effects [36] that may affect future antibody applications. A developing step of an indirect ELISA is using a conjugated peptide [37] such as biotinylated T14. This conjugation results in a consistent orientation and binding of the antigen to the plate, exposing the epitope, thereby enhancing signal, reducing background noise and reducing non-specific binding to the plate [38,39]. This improves reliability and reproducibility for further experiments and is an excellent standardized step for an ELISA.

The two conjugates used for T14 were BSA and Biotin on the N-terminus and were compared to unconjugated free T14 to assess the binding affinity with the selected mAb, THK-117. For native T14, a higher concentration of antibody was required, needing 2-fold dilutions and beginning at 240 µg/mL, compared to the conjugated peptides, which needed 3-fold dilutions starting from 10 µg/mL to produce a binding profile for characterization. This is likely due to the binding orientation of T14 and increased background noise in the absence of a conjugated peptide, highlighting the importance of using a conjugated peptide ELISA as a standardized approach. The low EC_50_ values of the conjugated T14 revealed a higher binding affinity compared to the unconjugated T14 [40]. These results indicate that THK-117 would likely be effective at low concentrations, consistent with its EC_50_ profile, and highlight the importance of ELISA-based approaches in quantitative antibody validation for therapeutic development.

## 5. Conclusions

This research demonstrates the rescue effects of THK-117 in reference to the T14 hypothesis, as well as the importance of antibody validation for Alzheimer’s disease treatment. THK-117 and T14 demonstrate a specific interaction, suggesting that THK-117 has the potential, if humanized, to modulate aberrant T14 activity and may represent a candidate for further therapeutic development. While in vivo studies would provide further translational validation, the present work focuses on establishing a structured in vitro and ex vivo antibody validation pipeline, which is a prerequisite for subsequent animal studies. Antibody therapeutic research would not be possible without a standardized validation approach to ensure preclinical efficacy translates into clinical success. This rigour is essential for advancing THK-117 from a research tool to an effective therapeutic candidate that could help mitigate Alzheimer’s disease pathology.

## Data Availability

The raw data generated during this study can be requested from the corresponding author upon reasonable request.

## Abbreviations

The following abbreviations are used in this manuscript:

AD	Alzheimer’s disease
α7 nAChR	Alpha-7 nicotinic acetylcholine receptor
pAb	Polyclonal antibody
ADA’s	Anti-drug antibody response
mAb	Monoclonal antibody
P7	Postnatal day 7
HPC	Hippocampus
PVDF	Polyvinylidene Fluoride
BSA	Bovine Serum Albumin
TBS	Tris-buffered saline solution
TBS-T	Tris-buffered saline solution + Tween-20
HRP	Horseradish Peroxidase
RT	Room Temperature
DMEM	Dulbecco’s Modified Eagle Medium
HS	Horse Serum
FBS	Fetal Bovine Serum
HHBS	Hanks Balanced Salt Solution
ELISA	Enzyme-linked immunosorbent assay
PBS-T	Phosphate-buffered saline + Tween-20
PBS	Phosphate-buffered saline
LSD	Least Significant Difference
SEM	Standard Error of Mean
CDR	Complementary-determining region
